# Characteristics Associated With Telemonitoring Use Among Patients With Chronic Heart Failure: Retrospective Cohort Study

**DOI:** 10.2196/43038

**Published:** 2023-10-18

**Authors:** Stefan L Auener, Simone A van Dulmen, Femke Atsma, Onno van der Galiën, Louise Bellersen, Roland van Kimmenade, Gert P Westert, Patrick P T Jeurissen

**Affiliations:** 1 IQ Healthcare Radboud Institute for Health Sciences Radboud University Medical Center Nijmegen Netherlands; 2 Strategy & Innovation Zilveren Kruis Achmea Zeist Netherlands; 3 Department of Cardiology Radboud University Medical Center Nijmegen Netherlands

**Keywords:** heart failure, telemonitoring, remote monitoring, eHealth, chronic heart failure, heart, disease, patient, self-management, prevention, utilization, Netherlands, hospital, treatment

## Abstract

**Background:**

Chronic heart failure (HF) is a chronic disease affecting more than 64 million people worldwide, with an increasing prevalence and a high burden on individual patients and society. Telemonitoring may be able to mitigate some of this burden by increasing self-management and preventing use of the health care system. However, it is unknown to what degree telemonitoring has been adopted by hospitals and if the use of telemonitoring is associated with certain patient characteristics. Insight into the dissemination of this technology among hospitals and patients may inform strategies for further adoption.

**Objective:**

We aimed to explore the use of telemonitoring among hospitals in the Netherlands and to identify patient characteristics associated with the use of telemonitoring for HF.

**Methods:**

We performed a retrospective cohort study based on routinely collected health care claim data in the Netherlands. Descriptive analyses were used to gain insight in the adoption of telemonitoring for HF among hospitals in 2019. We used logistic multiple regression analyses to explore the associations between patient characteristics and telemonitoring use.

**Results:**

Less than half (31/84, 37%) of all included hospitals had claims for telemonitoring, and 20% (17/84) of hospitals had more than 10 patients with telemonitoring claims. Within these 17 hospitals, a total of 7040 patients were treated for HF in 2019, of whom 5.8% (409/7040) incurred a telemonitoring claim. Odds ratios (ORs) for using telemonitoring were higher for male patients (adjusted OR 1.90, 95% CI 1.50-2.41) and patients with previous hospital treatment for HF (adjusted OR 1.76, 95% CI 1.39-2.24). ORs were lower for higher age categories and were lowest for the highest age category, that is, patients older than 80 years (OR 0.30, 95% CI 0.21-0.44) compared to the reference age category (18-59 years). Socioeconomic status, degree of multimorbidity, and excessive polypharmacy were not associated with the use of telemonitoring.

**Conclusions:**

The use of reimbursed telemonitoring for HF was limited up to 2019, and our results suggest that large variation exists among hospitals. A lack of adoption is therefore not only due to a lack of diffusion among hospitals but also due to a lack of scaling up within hospitals that already deploy telemonitoring. Future studies should therefore focus on both kinds of adoption and how to facilitate these processes. Older patients, female patients, and patients with no previous hospital treatment for HF were less likely to use telemonitoring for HF. This shows that some patient groups are not served as much by telemonitoring as other patient groups. The underlying mechanism of the reported associations should be identified in order to gain a deeper understanding of telemonitoring use among different patient groups.

## Introduction

Chronic heart failure (HF) is a major burden both in terms of health losses and financial expenditures. This progressive chronic disease affected more than 64 million people worldwide in 2017 [1]. As the leading cause of hospitalization for patients older than 65 years, HF puts significant pressure on the limited resources of health care systems, such as health care personnel and budgets [2]. Currently, many developed countries spend 1% to 2% of their health care budgets on this disease [3]. Furthermore, the prevalence of HF is expected to rise due to increased survival rates and an aging population [1]. Telemonitoring has often been proposed as an eHealth solution to reduce the number of admissions, which are a major cost driver in this population, as almost 1 in 4 patients is readmitted within 30 days [4,5]. While telemonitoring is not a homogeneous intervention, it is commonly defined as the measurement and transfer of physiological parameters through communication technologies to improve self-management and patient satisfaction and to reduce use of the health care system [6,7]. While there have been many studies on the topic of effectiveness, little is known about the actual use of telemonitoring among different hospitals or patient characteristics associated with telemonitoring use. Insight in the dissemination of this new technology among hospitals and patients may inform strategies for further adoption.

Previous studies have mainly focused on the effect of telemonitoring interventions on use of the health care system by patients with HF. While systematic reviews have shown ambiguous results [7,8], the number of studies showing significant reductions indicates that there are conditions under which telemonitoring can be an effective approach in reducing costs. In addition to the prevention of exacerbations, thereby reducing costs, other aims, such as increased quality of life [9,10], substitution of outpatient visits for titration of medication [11], and increased self-management [9,10,12], may be pursued. While many studies have aimed to investigate which benefits telemonitoring can potentially bring, little attention has been paid to which patients are the recipients of such benefits in the real world.

Previous studies that investigated the relationship between patient characteristics and eHealth use have had highly heterogeneous interventions and populations in research settings. These studies found that factors such as age, eHealth literacy, socioeconomic status (SES), and sex were associated with the use of eHealth [13-15]. While these findings may inform hypotheses and guide research toward areas of interest, generalizability to specific populations and interventions is largely unknown. Reiners et al [15] showed in a systematic review that these studies are heterogeneous regarding technologies used, geographical regions, and the diseases for which eHealth was deployed. Furthermore, most studies in this review described the acceptance and intended use instead of the actual use. Hence, the results from these studies may not be representative of the HF population and actual application of telemonitoring.

The research on telemonitoring use in HF populations is rather scarce and largely consists of qualitative research in an experimental setting [16]. These studies identified certain characteristics, such as sex [15], low SES [17], higher age [18], and lower health status [19], as potential barriers for the use of telemonitoring. While these qualitative results are specific for the HF population, information on the degree to which telemonitoring use is associated with these characteristics is lacking.

HF is more prevalent in older people with low SES [20] and low health status, and the barriers that these characteristics pose in the use of telemonitoring may be substantial. However, the actual use and the degree to which patient characteristics are associated with the use of telemonitoring for HF are unknown. Therefore, the aim of this study was to explore the adoption of telemonitoring and to quantitatively identify patient characteristics associated with the use of telemonitoring for HF. This information can be used to identify patient groups that are less likely to receive telemonitoring and develop specific implementation protocols for these patient groups.

## Methods

### Context

The study was situated in the Dutch health care system, which is characterized by universal social health insurance. All inhabitants are obliged to purchase health insurance, and it is prohibited to decline applicants (ie, there is open enrollment). The Dutch government is responsible for selecting the care that is insured through every health insurer by means of a basic care package. This package covers almost all curative inpatient and outpatient health care, with only certain exceptions, such as physiotherapy, dental care, and birth control, for which additional health care insurance can be purchased voluntarily. This system results in 99.8% of all inhabitants having health care insurance. Comparable to diagnosis-related groups (DRGs) for reimbursement, the Dutch health care systems uses diagnosis treatment combinations (DTCs) for hospital claims that are derived from registered care activities.

### Patient Selection

**Figure 1:**
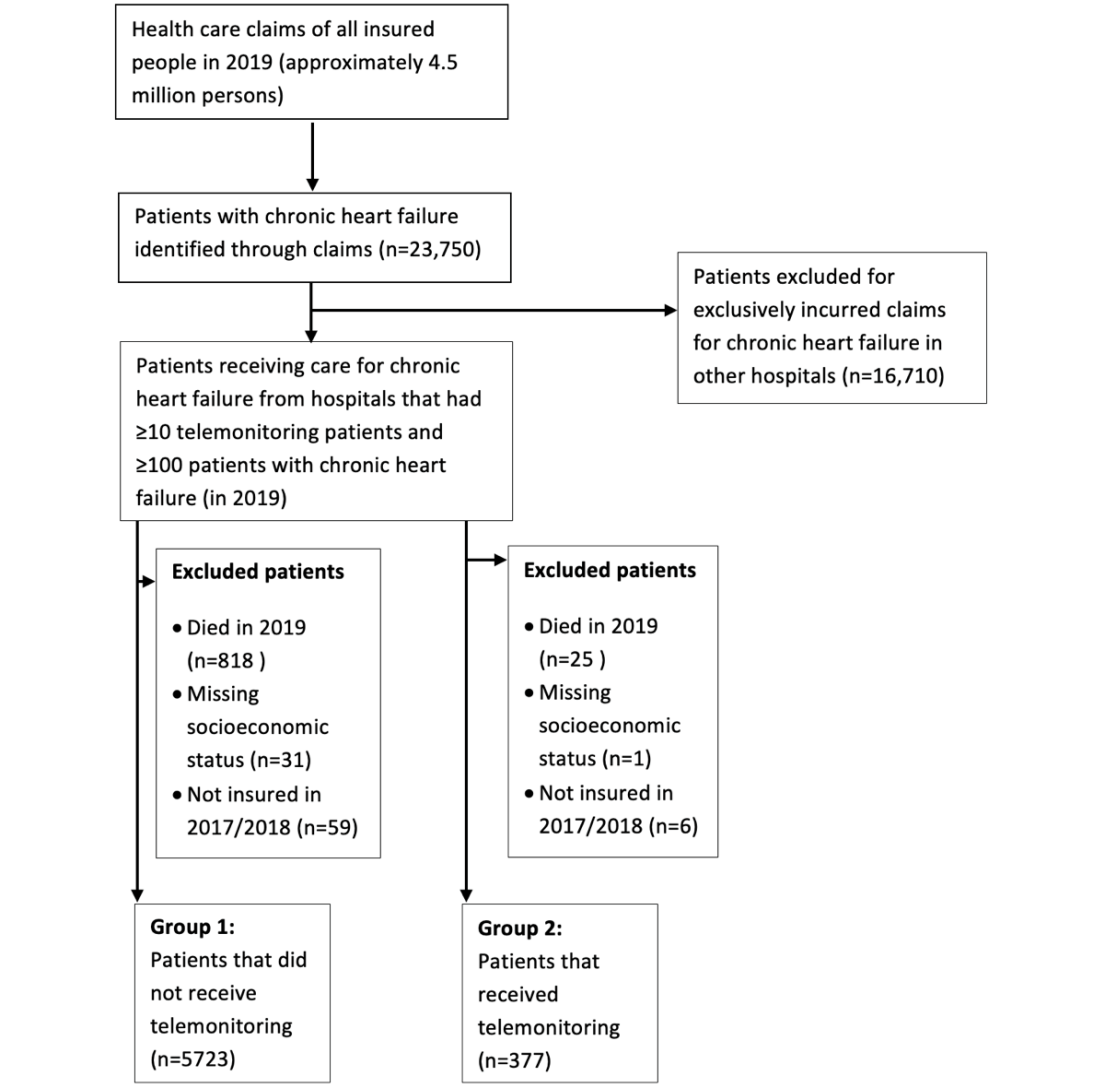
Flowchart of patient selection.

For this retrospective cohort study, we used routinely collected anonymized claim data from the largest Dutch health care insurance company, Zilveren Kruis Achmea. This health care insurance company covers 29% of the Dutch population (approximately 4.5 million persons), and its customers are representative of the Dutch population in terms of SES and age [21,22]. Patient selection was performed based on the DTCs described in Multimedia Appendix 1 by searching for DTC descriptions that contained the term “heart failure.” These DTCs cover all outpatient and inpatient care activities specifically for HF. Claim data and general information since January 1, 2017, were retrieved for patients that claimed at least one of these products between January 1, 2019, to December 31, 2019. To avoid small sample variance, we included only hospitals with a minimum of 10 patients with telemonitoring for HF and 100 patients with HF. Patients who died in 2019 were excluded to ensure complete follow-up. In addition, patients with missing postal codes and therefore missing SES, as well as patients that were not insured with Zilveren Kruis Achmea in the previous 2 years, were excluded from the analyses. Figure 1 shows how the data set for our analyses was created.

### Variables

Telemonitoring use was determined by identifying the care activity code for telemonitoring, defined as follows: “Remote monitoring of patients over a longer period of time as part of a treatment plan. The remote monitoring consists of the collection and interpretation of clinical data that is measured and transferred by the patients from their homes” [23]. The activity code does not prescribe an exact form and organization of the telemonitoring program, but health care insurers may require additional specifications for reimbursement. This activity code did not include CardioMEMS, an invasive telemonitoring system measuring intra-arterial pressure that differs substantially from noninvasive telemonitoring systems [24,25]. While the activity code for telemonitoring had been available prior to 2019, legislative requirements were changed in January 2019 to improve the possibilities for reimbursement of telemonitoring. The activity code did not directly lead to reimbursement, as hospitals had to make arrangements with health care insurance companies regarding compensation.

General information on patients comprised self-reported sex, date of birth and death, and postal code. The 4-digit postal code was used to assess neighborhood SES by using data from the Netherlands Institute for Social Research from 2016. These SES scores are based on multiple indicators for SES, such as percentage of residents with low education levels and average income, and are commonly used in health outcomes research in the Netherlands [26]. SES scores were divided in tertiles, and patients were assigned a high, average, or low SES based on their 4-digit postal code. Several indicators of health status were created, including the degree of multimorbidity, polypharmacy and excessive polypharmacy, and previous hospital HF treatment. An overview of the definition of all used variables is provided in Multimedia Appendix 1.

The degree of multimorbidity was derived by using pharmaceutical claims. Claim data on pharmaceutical use included the anatomical therapeutic chemical (ATC) class, which was used to identify diseases by Huber et al [27]. This allowed for a selection of 22 chronic diseases to be specifically identified from pharmaceutical claim data. Subsequently, the degree of multimorbidity was defined as the aggregated number of chronic diseases.

Polypharmacy and excessive polypharmacy were defined as having at least 5 and having 10 or more prescriptions for medication, respectively. We used the third level of the ATC classification system to determine the number of different medications. The level of the ATC class and cutoff levels for polypharmacy and excessive polypharmacy were based on the Dutch multidisciplinary guidelines for polypharmacy in older people [28]. Insurance claims do not indicate the disease stage in terms of New York Heart Association (NYHA) class or date of first diagnosis. Therefore, we created a variable, *previous HF treatment*, that indicated hospital treatments, including outpatient visits, for HF in 2017 or 2018.

### Analyses

First, we performed descriptive analyses to gain insight in the use of telemonitoring per hospital. We described the study population in terms of mean (SD) and number with proportion. All hospitals were classified as either hospitals delivering telemonitoring or hospitals not delivering telemonitoring. Thereafter, the number of unique patients per hospital was counted, and we determined whether they were telemonitoring users or not. Secondly, the GLIMMIX procedure in SAS Enterprise Guide (version 7.1; SAS Institute) was used to conduct a logistic multilevel analysis to explore the association between telemonitoring claims and the following variables: age, sex, SES, degree of multimorbidity, excessive polypharmacy, and previous HF treatment. A random intercept was introduced to account for the cluster effect within hospitals. Age and degree of multimorbidity were both defined as categorical variables, as linearity could not be assumed. Polypharmacy was not included in the model, as polypharmacy was a prerequisite for excessive polypharmacy, which introduced collinearity. Variables of interest were first solely included in the regression to obtain unadjusted estimates to allow for comparison to other studies. Thereafter, we performed multiple regression analysis including all variables simultaneously to obtain adjusted estimates. For both analyses, the outcome consisted of the presence of at least one activity code for telemonitoring. Statistical significance was specified as *P*<.05. The study is reported according to the STROBE (Strengthening the Reporting of Observational Studies in Epidemiology) checklist, which is provided in Multimedia Appendix 2 [29].

### Ethical Considerations

The study was performed according to the Declaration of Helsinki and reviewed by the medical ethical committee of the Radboud University Medical Center, Nijmegen (2019-5516). The committee determined that the study was exempt from the Dutch Medical Research Involving Human Subjects Act. The research was performed on anonymized data.

## Results

### Telemonitoring Use Among Hospitals

A total of 84 hospitals were identified as delivering HF care in 2019, with a total number of 23,750 patients with HF. The 84 hospitals included 10 independent treatment centers that delivered HF care. Of these, 63% (53/84) had no claims for telemonitoring, 17% (14/84) had telemonitoring claims for fewer than 10 (range 1-8) patients, 20% (17/84) had more than 10 patients on telemonitoring, and 29% (5/17) had more than 10% of their HF population on telemonitoring. Within these 17 hospitals, a total of 7040 patients were treated for HF in 2019, of whom 5.8% (409/7040) incurred a telemonitoring claim. The average percentage of the HF population on telemonitoring across these hospitals was 7.3% (SD 3.8%), ranging from 1% to 17%.

### Population Characteristics of Telemonitoring Users

After application of the exclusion criteria, a total of 6100 patients with HF remained for analysis, of whom 6.2% (377/6100) used telemonitoring. Table 1 shows descriptive statistics of the population used for analysis. Telemonitoring users were aged on average 68.8 (SD 10.5) years, and patients without telemonitoring had a mean age of 73.5 (SD 12.2) years. We found that 72.7% (274/377) of patients were male in the telemonitoring group and 54.4% (3115 /5723) of patients were male in the group without telemonitoring. Most patients in both the nontelemonitoring group (1830/5723, 32%) and telemonitoring group (181/377, 48%) had 3 or 4 chronic conditions. The vast majority of patients in both the telemonitoring group (293/377, 77.7%) and nontelemonitoring group (4178/5723, 73%) had polypharmacy. Furthermore, 70% (264/377) of patients in the telemonitoring group had received hospital treatment for HF in the previous 2 years. This percentage was 55.5% (3177/5723) in the group without telemonitoring.

**Table 1:** Characteristics of patients on January 1, 2019.

Characteristics		No telemonitoring (n=5723)	Telemonitoring (n=377)
Age (years), mean (SD)		73.5 (12.2)	68.8 (10.5)
**Age (years), n (%)**			
	18-59	748 (13.1)	70 (18.6)
	60-69	1016 (17.8)	113 (30)
	70-79	1901 (33.2)	137 (36.3)
	≥80	2058 (36)	57 (15.1)
**Sex, n (%)**			
	Male	3115 (54.4)	274 (72.7)
	Female	2608 (45.6)	103 (27.3)
Degree of multimorbidity, mean (SD)		3.1 (1.7)	3.2 (1.6)
**Degree of multimorbidity, n (%)**			
	0	333 (5.8)	22 (5.8)
	1-2	1784 (31.2)	99 (26.3)
	3-4	1830 (32)	181 (48)
	≥5	1776 (31)	75 (19.9)
Polypharmacy, n (%)		4178 (73)	293 (77.7)
Excessive polypharmacy, n (%)		1246 (21.8)	94 (24.9)
**Socioeconomic status, n (%)**			
	High	1922 (33.6)	113 (30)
	Middle	1903 (33.3)	134 (35.5)
	Low	1998 (33.2)	130 (34.5)
Previous treatment for chronic heart failure, n (%)		3177 (55.5)	264 (70)

### Patient Characteristics Associated With Receiving Telemonitoring

Table 2 shows the unadjusted and adjusted odds ratios (ORs) of our univariate and multiple logistic regression analyses. Statistically significant variables (*P*<.05) in the univariate analyses were age, sex, and previous hospital treatment. These variables remained significant in the multiple regression analyses. Patients older than 80 years had significantly (*P*<.001) lower odds of receiving telemonitoring (adjusted OR 0.30, 95% CI 0.21-0.44) than patients in the reference group (age category 18-59 years). Sex was significantly (*P*<.001) associated with telemonitoring, with male patients having an adjusted OR of 1.90 (95% CI 1.50-2.41) for receiving telemonitoring compared to female patients. Patients with previous HF treatment had an OR of 1.76 (95% CI 1.39-2.24) for receiving telemonitoring (*P*<.001). The degree of multimorbidity, SES, and excessive polypharmacy were not significantly associated with receiving telemonitoring.

**Table 2:** Odds ratios for using telemonitoring derived from univariate and multiple logistic regression analyses.

			Univariate regression				Multiple regression		
			Unadjusted OR^a^ (95% CI)		*P* value	Adjusted OR (95% CI)			*P* value
**Age group (years)**									
	18-59 (reference)	1.00		N/A^b^		1.00		N/A	
	60-69	1.25 (0.91-1.72)		.18		1.20 (0.87-1.67)		.27	
	70-79	0.78 (0.57-1.06)		.12		0.76 (0.55-1.04)		.09	
	≥80	0.30 (0.21-0.44)		<.001		0.30 (0.21-0.44)		<.001	
**Sex**									
	Female (reference)	1.00		N/A		1.00		N/A	
	Male	2.18 (1.72- 2.75)		<.001		1.90 (1.50-2.41)		<.001	
**Degree of multimorbidity**									
	0 chronic diseases (reference)	1.00		N/A		1.00		N/A	
	1-2 chronic diseases	0.81 (0.50-1.32)		.40		0.80 (0.49-1.31)		.38	
	3-4 chronic diseases	1.09 (0.69-1.73)		.72		1.10 (0.68-1.79)		.70	
	≥5 chronic diseases	0.96 (0.58-1.58)		.87		0.87 (0.49-1.54)		.63	
**Socioeconomic status**									
	High (reference)	1.00		N/A		1.00		N/A	
	Medium	1.04 (0.79-1.38)		.77		1.04 (0.78-1.38)		.80	
	Low	0.86 (0.66-1.14)		.28		0.84 (0.63-1.11)		.22	
**Excessive polypharmacy**									
	No (reference)	1.00		N/A		1.00		N/A	
	Yes	1.16 (0.91-1.48)		.24		1.27 (0.92-1.76)		.15	
**Previous heart failure treatment**									
	No (reference)	1.00		N/A		1.00		N/A	
	Yes	1.88 (1.49-2.36)		<.001		1.76 (1.39-2.24)		<.001	

^a^OR: odds ratio.

^b^N/A: not applicable.

## Discussion

### Principal Findings

This study explored the use of telemonitoring for HF in the Netherlands and determined which patient characteristics were associated with telemonitoring use. Our results indicate that there was high variation in telemonitoring use for HF patients between hospitals in the Netherlands in 2019. The use of telemonitoring was associated with several patient characteristics. Age, sex, and previous hospital treatment for HF were significantly associated with using telemonitoring. Degree of multimorbidity, excessive polypharmacy, and SES were not statistically significant associated with the use of telemonitoring for HF.

### Adoption of Telemonitoring

Our findings suggest that the uptake of telemonitoring for patients with HF seemed to be limited, at least up to 2019. This indicates that the use of telemonitoring was likely mostly restricted to pilot tests and the early adoption phase. A survey among Dutch HF clinics suggested that upward of 20% of patients with HF may be able to use telemonitoring [30]. Our analyses showed that only one hospital came close to this, with 17% of HF patients receiving telemonitoring. Furthermore, within hospitals that deployed telemonitoring, the average percentage of all patients with HF using telemonitoring was less than 6%. This shows that adoption is not only limited due to limited diffusion among hospitals but also due to limited adoption within hospitals.

Whereas the uptake of telemonitoring in general had been relatively slow up to 2019 in the Netherlands [31], the COVID-19 pandemic has accelerated the adoption of digital health care services such as telemonitoring [32,33]. While there are no exact data for telemonitoring specifically, the number of eHealth claims for patients with cardiac diseases increased more than 300% in 2020 compared to 2019 in the Netherlands [33]. Large increases for eHealth use were also found in other countries, such as the United States, with an increase of 154% in telehealth visits [34].

### Patient Characteristics Associated With Telemonitoring

#### Association With Sex

An important finding of this study is that being female was independently associated with lower odds of receiving telemonitoring. Differences between sexes have received increasingly more attention, both within cardiology and in digital health. Within cardiology, previous research has shown that there are meaningful differences between the sexes in clinical characteristics and therapeutic responses to different treatments [35]. A possible explanation for the difference is the higher prevalence of HF with preserved ejection fraction (HF-pEF) among female patients, which has limited treatment options compared to HF with reduced ejection fraction (HF-rEF) [36]. Fewer medical treatment options may translate into less need for remote monitoring of titration of medication [11].

Within digital health, there are significant differences between men and women concerning the use of new technologies. The findings of these studies are conflicting, with some studies finding higher but most studies suggesting lower use of eHealth among women [37-39]. A German study from 2013 found that women generally were less willing to use telemonitoring. Moreover, this willingness further decreased with age, as younger women generally had greater computer literacy [38]. While the Netherlands is one of the most digitalized countries in the EU, there is still a large group with low digital skills, among whom women are overrepresented [40]. Other possible explanations may be a lack of social support, as women are more often widowed [41], or implicit biases of health care professionals [42]. While our study shows that there is a difference in use of telemonitoring between sexes, it does not provide information on the reasons for this, and one should be careful to draw conclusions regarding the underlying mechanisms of these differences.

#### Association With Age

The odds for receiving telemonitoring were lower for patients in older age categories. This may signal that there are certain barriers for this population in the use of telemonitoring. This is important information, as telemonitoring is often proposed as a solution for high health care costs and shortages in labor markets. Yet a substantial and expanding group of older patients are currently less likely to use this intervention. This may be due to lower self-efficacy and digital literacy within this population [43]. Another reason may be a lack of evidence for a positive effect of telemonitoring in older people, as most studies on telemonitoring are performed with study populations with an average age younger than 70 years [44,45].

#### Association With SES

SES was not significantly associated with telemonitoring use. This is not consistent with most international studies, which show that patients with a high SES are first to adopt and benefit from new technologies [15,46,47]. A possible explanation for this discrepancy may be our method of assessing SES based on postal code rather than a more accurate individual status. However, our method is commonly used in studies in the Netherlands and has shown to predict health outcomes such as health care costs [48], adverse birth outcomes [26], and survival rates for stomach cancer [49].

#### Association With Health Status

Multimorbidity and excessive polypharmacy were not associated with use of telemonitoring. Patients with a higher degree of multimorbidity and excessive polypharmacy have higher odds of incurring high costs and are therefore an important target population for interventions aiming to reduce costs, such as telemonitoring [5]. Systematic exclusion of these patients would likely limit the reduction in health care use that telemonitoring can potentially offer. However, our study showed that this population is neither specifically included nor excluded in current telemonitoring programs.

The association of previous hospital treatment for HF and telemonitoring use may indicate that telemonitoring is used for advanced forms of heart failure and may suggest that health care professionals expect more benefit in these patients, which is congruent with many studies limiting inclusion of patients to NYHA class II or III [7]. An alternative hypothesis is that the needs of patients regarding kind of contact and support change over time, comparable to how other needs change during disease progression [50].

### Implications

Most hospitals that deployed telemonitoring had relatively few patients using telemonitoring for HF in 2019. While the percentages found in this study will likely now no longer be the same due to accelerated adoption during the COVID-19 crisis [33], our results show that significant uptake of telemonitoring is not self-evident, as telemonitoring had already been used for years. Future research should therefore focus on both diffusion among hospitals as well as further adoption within hospitals. While there have been studies on enablers and barriers, such as funding and national coordination, there is a need for converting those enablers and barriers into actionable guidelines that support nationwide upscaling [51].

As there can be multiple reasons for the differences we found in the use of telemonitoring, additional research is needed on how these differences come into existence and how stakeholders should deal with this. This is important, as unexplained differences in use may indicate suboptimal use [52]. There are likely to be underlying mechanisms and unobserved confounders, such as self-efficiency, social support, IT competency, and digital literacy, which we were not able to capture using claim data [16]. Future research should therefore aim to gain insight into the mechanisms that result in lower telemonitoring use among certain patient groups. Information on these working mechanisms can be used to inform guidelines for optimal telemonitoring use and to adapt telemonitoring programs to patients’ needs.

Our results suggest that eHealth applications such as telemonitoring are not used evenly across sociodemographic categories. These differences in use may occur due to a variety of reasons, as we described previously. Skewed use among certain patient groups may induce inequality of health outcomes. Health care providers, researchers, and policymakers should keep the possible occurrence of such inequalities in mind when introducing innovations, and they should therefore not only investigate the benefits on an aggregated level. Moreover, they should also monitor and reflect on which patient groups may be left behind and which are in need of an additional or different approach.

### Strengths and Limitations

A major strength of the study is the use of claim data from the largest health care insurance company in the Netherlands, with a market share of 29%. This allowed us to explore several associations between patient characteristics and telemonitoring use in a real-world setting that included virtually all hospitals in the Netherlands. Furthermore, as the population of Zilveren Kruis Achmea is representative of the Dutch population with HF, our findings have high generalizability within the Dutch population.

Certain limitations should be taken into account when interpreting the data. First, the data used here were collected before the COVID-19 pandemic, as more recent data were not available. Since then, digital adoption has seen enormous acceleration, and the reported use is no longer representative of current use [32,53]. However, the use of data collected prior to COVID-19 might also be a strength, since the decisions by health care providers to use telemonitoring during the COVID-19 crisis were not based on a normal situation in which patients receive usual care [54].

Second, as a consequence of using claim data, telemonitoring status was dependent on the hospital registering telemonitoring activities. Some hospitals may not have registered telemonitoring activities but may still have offered a form of telemonitoring to their patients. This may have resulted in an underestimation of the number of hospitals deploying telemonitoring. Since our regression analyses were based on the populations within telemonitoring hospitals, the associations we found between telemonitoring use and patient characteristics were not affected.

Third, telemonitoring use was defined as a dichotomous outcome. Due to the type of information retrievable from health care claim data, it was not possible to retrieve the intensity of, and patient adherence to, telemonitoring. The reported associations are therefore mainly informative on the initial use of telemonitoring rather than continued use.

### Conclusion

The use of telemonitoring for HF in the Netherlands was limited up to 2019, and our results indicate that there is large variation among hospitals. A lack of adoption is therefore not only due to a lack of diffusion among hospitals but also due to a lack of scaling up within hospitals that already deploy telemonitoring. Future studies should therefore focus on both kinds of adoption and how to facilitate these processes. Older patients, female patients, and patients with no previous hospital treatment were less likely to use telemonitoring for HF. This shows that some patient groups are not as well served by telemonitoring as other patient groups. The underlying mechanism of the reported associations should be identified in order to gain a deeper understanding of telemonitoring use among different patient groups.

## Appendix

Multimedia Appendix 1 Definition of variables based on claim data.

Multimedia Appendix 2 STROBE (Strengthening the Reporting of Observational Studies in Epidemiology) checklist.

## Abbreviations

ATC: anatomical therapeutic chemical
DRG: diagnosis-related group
DTC: diagnosis treatment combination
HF: heart failure
HF-pEF: heart failure with preserved ejection fraction
HF-rEF: heart failure with reduced ejection fraction
NYHA: New York Heart Association
OR: odds ratio
SES: socioeconomic status

## Data Availability

The data sets generated during and/or analyzed during this study are not publicly available due this data containing competitively sensitive information but are available from the corresponding author on reasonable request.
