# Supplementary material for: Characteristics Associated With Telemonitoring Use Among Patients With Chronic Heart Failure: Retrospective Cohort Study
Source: J Med Internet Res. 2023 Oct 18;25:e43038. doi: 10.2196/43038 (PMC10620630; doi:10.2196/43038)
Supplement: Multimedia Appendix 1 [file jmir_v25i1e43038_app1.docx]

Multimedia appendix 1: Definition of variables based on claim data

| **Variable** | | **Definition/ Source** |
| --- | --- | --- |
| Age | | Derived from insurance file |
| Sex | | Derived from insurance file |
| Socioeconomic status | | Derived from Postal ZIP code |
| Degree of multimorbidity | | Sum of chronic diseases |
| Polypharmacy | | Having at least 5 chronic medications at the third level of ATC-class |
| Excessive polypharmacy | | Having at least 10 chronic medications at the third level of ATC-class |
| Previous hospital treatment for HF | | A claim for HF in 2017 or 2018 |
| HF claim diagnosis treatment codes | 099899045 | Diagnostics/surgery and/or more than 2 outpatient clinic visits/remote consultations in chronic heart failure |
|  | 099899046 | A maximum of 5 day treatments and/or nursing days for chronic heart failure |
|  | 099899066 | 1 or 2 outpatient clinic visits/remote consultations for chronic heart failure |
|  | 099899067 | More than 28 day treatments and/or nursing days for chronic heart failure |
|  | 099899068 | 6 to 28 day treatments and/or nursing days for chronic heart failure |
|  | 099899024 | a maximum of 5 day treatments and/or nursing days in acute heart failure |
|  | 099899049 | More than 28 day treatments and/or nursing days in case of acute heart failure |
|  | 099899050 | 6 to a maximum of 28 day treatments and/or nursing days for acute heart failure |
| Telemonitoring care activity code | 039133 | This care activity is registered for remote monitoring of a patient over a longer period of time in the context of a treatment plan. Monitoring takes place by collecting and  interpreting clinical data that are collected and transmitted by the patient from their home. |
| **Identifying chronic diseases to assess degree of multimorbidity** | | **ATC classification** |
| Acid related disorders | | A02 |
| Bone diseases (osteoporosis) | | M05 |
| Cancer | | L01 |
| Cardiovascular diseases (including hypertension) | | B01AA, B01AC, C01, C04A, C02, C07, C08, C09 |
| Dementia | | N06D |
| Diabetes mellitus | | A10A, A10B, A10X |
| Epilepsy | | N03 |
| Glaucoma | | S01E |
| Gout, Hyperuricemia | | M04 |
| HIV | | J05AE, J05AG,J05AR |
| Migraines | | N02C |
| Pain | | N02A,N02B |
| Parkinson’s disease | | N04A |
| Psychological disorders (sleep disorder, depression | | N05B, N05C, N06A |
| Psychoses | | N05A |
| Respiratory Illness (asthma, COPD) | | R03 |
| Rheumatologic conditions | | M01, M02, L04AA, L04AB |
| Thyroid disorders | | H03A |
| Tuberculosis | | J04A |
